# A Mixed Methods Study of Functioning and Rehabilitation Needs Following COVID-19

**DOI:** 10.3389/fresc.2021.710410

**Published:** 2021-08-31

**Authors:** Tina Backmann, Thomas Maribo, Ann-Dorthe Zwisler, Jesper Rømhild Davidsen, Nina Rottmann

**Affiliations:** ^1^REHPA, The Danish Knowledge Centre for Rehabilitation and Palliative Care, Odense University Hospital, Nyborg, Department of Clinical Research, University of Southern Denmark, Odense, Denmark; ^2^Department of Public Health, Centre for Rehabilitation Research, Aarhus University, Aarhus, Denmark; ^3^DEFACTUM, Central Denmark Region, Aarhus, Denmark; ^4^South Danish Center for Interstitial Lung Diseases (SCILS), Department of Respiratory Medicine, Odense University Hospital, Odense, Denmark; ^5^Odense Respiratory Research Unit (ODIN), Department of Clinical Research, University of Southern Denmark, Odense, Denmark; ^6^Department of Psychology, University of Southern Denmark, Odense, Denmark

**Keywords:** COVID-19, rehabilitation, persistent symptoms, functioning, disability, quality of life, mixed methods, ICF

## Abstract

COVID-19 can lead to a long-term loss of functioning, which may affect activities and participation in daily living in various ways. The extent and characteristics of post-COVID-19 persistent symptoms are currently being studied extensively worldwide. The purpose of this exploratory study is to explore functioning and rehabilitation needs among persons with self-reported disability following COVID-19. This mixed methods study is based on data from patient-reported outcome measures (PRO), tests of body functions, visual drawings and focus groups among persons with self-reported disability after having suffered from COVID-19. PRO covered quality of life, activity and participation. Tests of body functions targeted strength and endurance. Focus groups and visual drawings elaborated on how post COVID-19 persistent symptoms affected functioning, activities and daily living. Data was collected in August and September 2020. The study sample consisted of 11 women, nine men, aged 35–79 years. Self-reported PRO data showed low quality of life and disability among the participants primarily related to fatigue, energy and drive, breathing and concentration. Tests of body functions showed low strength in lower extremities but otherwise no striking limitations on a group level. Analysis of the focus groups generated the following four themes: (1) *Persistent symptoms*, particularly in regards to concentration, memory, lack of energy, fatigue and headaches. (2) *Balancing activities* in daily living with fluctuating symptoms. (3) *Uncertainty and Powerlessness*, which included a need for directional guidance in order to regain functioning and unmet needs regarding further clinical assessment of persistent symptoms, referral to rehabilitation and returning to work. (4) *Hope* associated with the experiences of recovery - and for the future. This study highlights that persons with persistent symptoms after COVID-19 may experience a range of limitations in their daily living. This points toward a need for individual assessment and guidance to tailor relevant rehabilitation.

## Introduction

The corona virus disease 2019 (COVID-19) outbreak and pandemic has affected a large number of persons worldwide, and a significant number of persons have experienced different degrees of illness. In 2021 the number of confirmed infections with the virus, SARS-CoV-2, is still steadily rising counting nearly 150 million cases and over 3 million deaths at the end of April 2021 (1).

During the pandemic it has become clear that some persons experience long term sequelae or “post COVID-19 persistent symptoms,” as termed by The World Health Organization (2). International systematic literature reviews based on the present and previous pandemics (SARS and MERS) show that persons who have been ill with viral disease can experience different challenges in relation to functioning and activities of daily living (3, 4). In addition, preliminary knowledge shows that persistent symptoms including fatigue, headache, dyspnoea and myalgia are highly prevalent among persons who have suffered from COVID-19 and that 80% experience one or more persistent symptoms (4, 5). However, we do not know the full extent of consequences of COVID-19, their impact on daily living and the overall need for rehabilitation, yet. The list of potential post-COVID-19 symptoms is long and studies worldwide are steadily uncovering the long-term consequences in further detail. Although persons with longer and more severe courses of illness may have an increased need for rehabilitation, recent research shows that persons with short courses also present with persistent disability (4, 6, 7). A large group of persons are therefore likely to need evidence based post-COVID-19 rehabilitation on both general and specialized level to also accommodate those with severe disability (8–10).

Healthcare systems worldwide have been struggling to adapt and manage both safety precautions and the treatment of many patients with an unknown life-threatening illness. In the same way, rehabilitation of persons with post COVID-19 persistent symptoms has represented unexplored territory. It is thus necessary both to describe the type and frequency of typical disability and rehabilitation needs following COVID-19, and to understand persons' experience of COVID-19-related persistent symptoms in their daily living. This knowledge is required to tailor disease specific rehabilitation. Such knowledge can also create a basis to evaluate to which extent existing knowledge from other illnesses can be part of the foundation for post COVID-19 rehabilitation interventions. To illuminate these areas, knowledge from different research methods must be used and combined: Quantitative methods can be used to describe the type and frequency of present disabilities and rehabilitation needs. Qualitative research methods can help us gain an in-depth understanding of the experience of COVID-19-related persistent symptoms in daily living.

The purpose of the present exploratory study is to uncover functioning and rehabilitation needs among persons with self-reported disability following COVID-19.

## Materials and Methods

The study was conducted as an explorative mixed methods study of persons who were ill with COVID-19 during spring and summer of 2020. The study is based on a convergent parallel design, in which the quantitative and the qualitative data are collected and analyzed concurrently. The results are then related to each other and interpreted (11).

The quantitative data include both patient-reported outcome measures (PRO) and tests of body functions and the qualitative data consist of focus groups including visual drawings.

Throughout this article, the ICF terminology is used according to the WHO standards (12).

### Setting and Organization

The study was conducted at REHPA, The Danish Knowledge Center for Rehabilitation and Palliative Care. The center is organized and imbedded in both a University and hospital setting. Some of the research at REHPA is conducted in a research clinic, where study participants can stay during group-based courses with different research purposes.

The study was carried out as part of the centre's efforts and research activities around COVID-19. The study participants participated in a course containing both clinical activities, such as physical training and workshops on dealing with symptoms, and research-related activities with focus on post-COVID-19 rehabilitation needs. Two courses of 4 days each were conducted in August and September 2020.

The study participants were invited through various media and patient organizations. In addition, information about the study was available online on REHPA's website, and written information material was sent to hospitals who were in contact with COVID-19 patients.

The participants were referred by a general practitioner or hospital physician through a referral form with medical information about the course of illness. In addition, the participants completed a personal electronic application form and gave their written consent to participate in the study.

A steering committee and an advisory group were set up to ensure quality in the COVID-19 studies at REHPA. The steering committee primarily contributed with guidance on the direction and aims of the studies. The advisory group primarily contributed with knowledge that ensured the quality of specific methods. The groups included researchers and clinicians as well as persons with post COVID-19 persistent symptoms.

### Inclusion Criteria

The research clinic had 10 men and 14 women referred of whom two withdrew their referral as they no longer experienced symptoms and two did not meet the inclusion criteria leaving 20 persons in the study. Persons who had suffered from COVID-19 and had self-perceived rehabilitation needs were included in the study. In the first rehabilitation course, only persons who had been hospitalized with COVID-19 were included. However, as the growing experience with COVID-19 indicated that persons, who had not been hospitalized, also might experience persistent symptoms, we expanded the inclusion criteria to also include persons who had not been hospitalized in the second course. Participants had to be able to care for themselves regarding hygiene, meals etc. It was prioritized to include corresponding to an equal gender distribution. Being an explorative study, this selection was made in order to assemble a diverse group.

### Data Collection

Data were collected through PRO, tests of body functions and focus groups including visual drawings.

#### PRO

The PRO data were collected before the participants arrived at the research clinic. Electronic questionnaires were sent out 3 weeks before the rehabilitation course. PRO measures included The Post COVID-19 Functional Status Scale (PCFS) and the REHPA scale as measures of disability, and the EuroQoL 5-dimensions 5-level (EQ-5D-5L) scale as a measure of quality of life. Sociodemographic information and information about the specific course of illness were included in the electronic questionnaires.

The REHPA Scale of Rehabilitation needs is inspired by the *National Comprehensive Cancer Networks Distress Thermometer and Problem List* (13) and developed at Dallund Rehabilitation center (14). It is used widely in cancer rehabilitation in Denmark but is not validated. The REHPA scale consists of a numeric ranking scale from 0 to 10, on which higher scores symbolize being far from living the life the participants wish and are able to live following COVID-19.

In addition to the numeric ranking scale, participants mark self-perceived causes of their loss of functioning on a 84 item list within the overarching areas *Practical issues, Work or school related issues, Family issues, Psychological issues, Physical issues* and *Spiritual or religious issues*. The participants mark the items they perceive being the ones preventing them from living life as they wish.

PCFS is an ordinal COVID-19 specific status scale used to measure the impact of disability within activity and participation in regards to daily living. This scale does not differentiate between underlying causes (15). The scale ranges from 0 to 5. Higher score indicates greater degree of restrictions.

The EQ-5D-5L includes a visual analog scale, on which participants indicate their self-rated health (16). The scale ranges from 0 to 100, where 100 equals the best imaginable health. In addition, the EQ-5D-5L has five descriptive dimensions: *Mobility, Self-care, Usual activity, Pain/discomfort* and *Anxiety/depression*. Each dimension has five levels ranging from *no problems* to *extreme problems*.

#### Tests of Body Functions

In this study we chose to explore the elements of body functions that cover strength and endurance. Tests of strength and endurance were conducted during the rehabilitation course as further basis to describe potential impairments within these domains. As COVID-19 in some areas resembles known respiratory illnesses, it is meaningful to use existing generic and disease-specific tools that are already being used in the rehabilitation of persons with, for example, COPD (17, 18) to assess rehabilitation needs. The present study included the 6-min walk test (6 MWT), the 30-s Sit-To-Stand test (30s-STS) and test of hand grip strength (HGS) measured with dynamometer. The 6 MWT measures the distance (6 MWD) in meters as an indirect surrogate measure of endurance.

The HGS test measures isometric grip strength (in kilograms) as a surrogate measure of strength in the upper extremities.

The 30 s-STS measures the number of times a person can rise from sitting position in 30 s as a surrogate measure of strength in the lower extremities (19, 20). In addition, height and weight was measured in order to calculate reference values for 6 MWT (21).

#### Focus Groups Including Visual Drawings

Four focus groups were facilitated by the first author of this article (TBA) (22).

TBA and last author developed the interview guide. The guide contained instructions for the interviewer on how to introduce the group session. The questions in the guide focused on facilitating dialogues about impairments and disability during and after COVID-19 with questions regarding:

- The experience of being ill
- Returning to daily living after illness
- Thoughts and experiences on how rehabilitation could bring the wished life within reach

The focus groups contained between 4 and 6 participants where the participants were divided so each group contained both men and women. The focus groups lasted for 75 min each.

TBA introduced the groups to the themes of the interview guide and invited the participants to have an open-minded dialogue with each other about these themes, giving room for different experiences and opinions. During the focus groups the participants were encouraged to elaborate experiences e.g., in regards to how certain symptoms affected activities and participation. In each group, the participants introduced themselves and then spent 5 min on a reflection task, where they drew a timeline on their symptoms from the acute phase of illness to present time. These visual drawings were inspired by the work of Carfì et al. and a Danish taskforce about the journey of being a patient (23, 24). The drawings were the starting point for the dialogue in the focus group.

### Data Analysis

Sociodemographic background information, PRO data and results from the tests of body functions were analyzed descriptively. Categorical data are presented as numbers and percentages. Continuous variables are presented as medians showing interquartile ranges (IQR) and the total range of scores. Tests of body functions were divided into gender groups. Data is only shown for subgroups larger than five participants to ensure anonymity. This meant that several subcategories on the items concerning education, occupation and month of diagnosis had to be merged.

All focus groups were recorded and transcripts formed the data for analysis. Thematic analysis, with a focus on meaning and participants' experiences, was conducted by TBA (25). The transcripts were coded and divided into overarching themes and sub-themes to identify patterns in the participants' dialogues. Transcripts were worked through several times in order to compare the data, refine coding, and synthesize themes. Themes and subthemes were listed and paired with quotes from across the transcripts.

### Ethics and Data Protection

All participants received oral and written information and gave written informed consent to participate. The study was approved and registered by the Region of Southern Denmark: Journal no. 20/30702.The REHPA-database was approved by the Danish Data Protection Agency and approved and registered by the Region of Southern Denmark: Journal no. 18/27843. The Regional Committee on Health Research Ethics for Southern Denmark assessed that the study was not notifiable: Case number 20202000, no. 122

## Results

### Quantitative Data

Of the 20 participants, 55% were women. The majority (80%) had been diagnosed with COVID-19 in March and April 2020. Additional sociodemographic characteristics of participants are presented in Table 1.

**Table 1:** Participant characteristics (*N* = 20).

**Characteristic**	***N* (%)**	**Median (IQR)**	**Range**
**Gender**			
Women	11 (55)		
Men	9 (45)		
Age in years		51.5 (46–68.5)	35–79
**Education**			
<3 years of education or no education exceeding primary school (6–16 years of age)	9 (45)		
≥3 years of education following primary school	11 (55)		
**Occupational status**			
Retired	7 (35)		
Full- or part time employment	9 (45)		
On sick leave	4 (20)		
**Month of COVID-19 diagnosis**			
03–04/2020	16 (80)		
05–06/2020	4 (20)		
**Admitted to hospital due to COVID-19**			
Yes	15 (75)		
No	5 (25)		
Days admitted to hospital (*n* = 15)		23 (10–30)	1–58

*IQR, interquartile range*.

Participants' PRO-data are presented in Tables 2, 3. Participants reported a range of physical-, psychological- and work- and school related issues as frequent causes to loss of functioning (Table 2). The median score on the PCFS and the EQ-5D-5L were 2 and 60, respectively (Table 3). Tests of body functions are presented in Table 4. To differentiate the results these are categorized into gender groups.

**Table 2:** REHPA scale of rehabilitation needs (*N* = 19).

	**Median (IQR)**	**Range**
**REHPA-scale**	**7 (3–9)**	**3–9**
**Area**	**Item**	***n* (%)**
Practical issues		<5
Work- or school related issues	Own expectations	6 (30)
Psychological issues	Worried	9 (45)
Physical issues	Headache	8 (40)
	Vertigo	7 (35)
	Balance	8 (40)
	Fatigue	16 (80)
	Exhaustion	13 (65)
	Memory	9 (45)
	Concentration	10 (50)
	Impaired mobility	5 (25)
	Decreased muscle strength	8 (40)
	Muscle- and joint pain	8 (40)
	Breathing	11 (55)
	Paraesthesia	5 (25)
Family issues		0
Spiritual or religious issues		0

*IQR, interquartile range*.

**Table 3:** Scales of functioning and health related quality of life (*N* = 20).

		***N* (%)**	**Median (IQR)**	**Range**
PCFS			2 (2, 3)	1–4
EQ-5D-5L			60 (50–65)	15–75
**EQ-5D-5L dimensions**				
Mobility	No problems	11 (55)		
	Problems	9 (45)		
Self-care	No problems	15 (75)		
	Problems	5 (25)		
Usual activity	No problems	0 (0)		
	Problems	20 (100)		
Pain/discomfort	No problems	3 (15)		
	Problems	17 (85)		
Anxiety/ depression	No problems	6 (30)		
	Problems	14 (70)		

*IQR, interquartile range; PCFS, post COVID-19 functional status scale; EQ-5D-5L, EuroQuol 5D five level scale*.

**Table 4:** Tests of body functions (*N* = 20).

	**Women (*****n*** **=** **11)**		**Men (*****n*** **=** **9)**	
	**Median (IQR)**	**Range**	**Median (IQR)**	**Range**
Age, median (IQR)	48 (44–52)	35–66	72 (55.5–75)	38–79
30 s-STS, median (IQR)	14 (12–24)	7–30	16 (12–18.5)	11–31
**HGS, median (IQR)**
Right	33.1 (27.0–35.0)	23.9–41.8	37.6 (28.1–41.75)	24.5–44.8
Left	30.1 (26.2–36.1)	19.5–38.4	37.0 (30.7–44.0)	29.1–48.6
**6 MWD, median (IQR)**
Distance (meters)	510 (450–552)	400–623	565 (442.5–587.5)	378–630
Percent of reference	95.5 (82.7–103.3)	73.7–119.3	95.5 (83.3–115.7)	80.1–116.5

*IQR, Interquartile range; 30 s-STS, 30 second Sit To Stand test; HGS, hand grip strength; 6 MWD, 6-minute walk distance*.

### Qualitative Data

The visual drawings were the starting point for the dialogues in the focus groups. One participant did not complete the drawing task, but all 20 participated in the following discussion.

**Figure 1:**
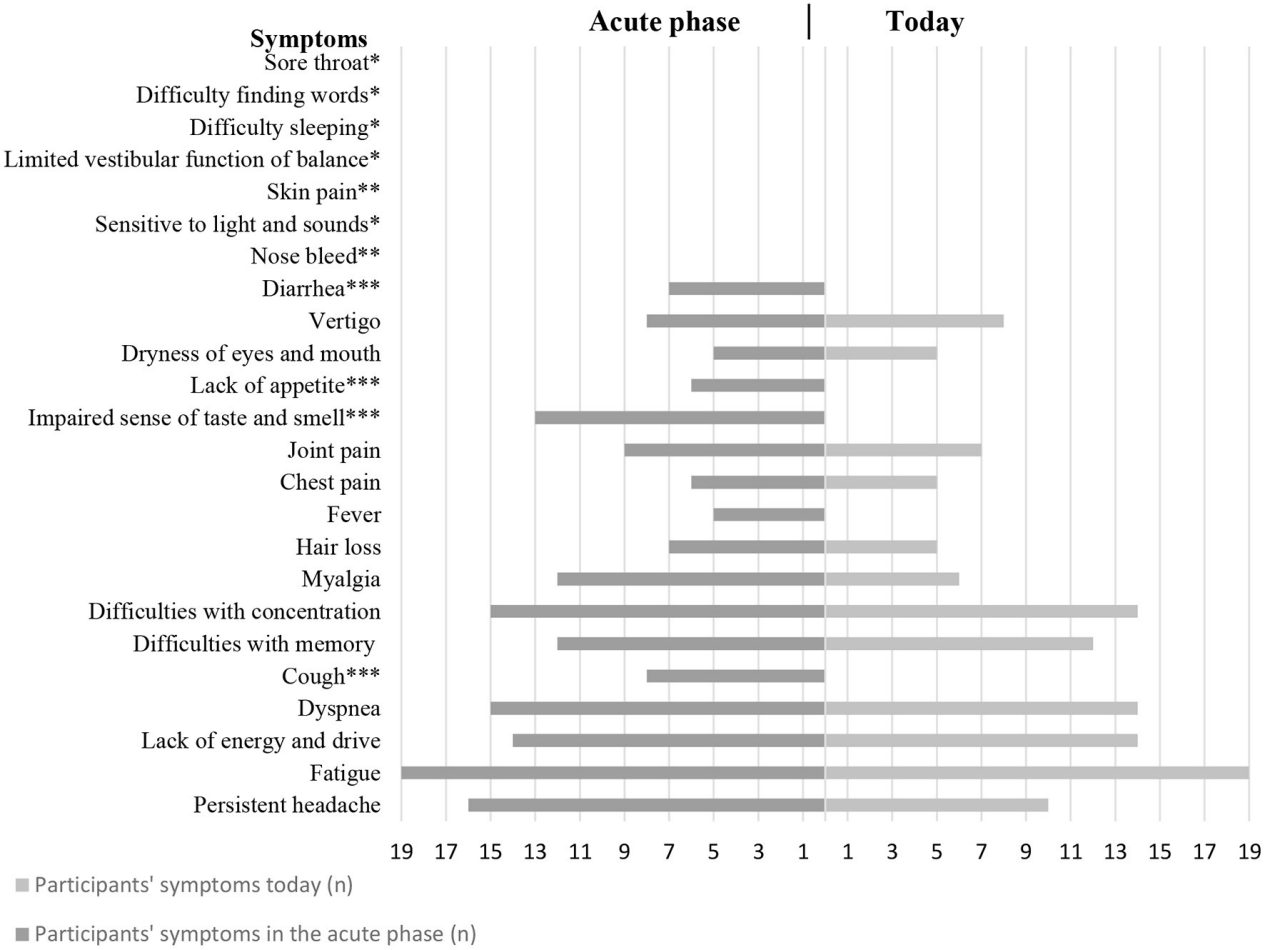
Overview of symptoms marked on the timeline drawings in the focus groups (*N* = 19). * <5 participants experienced these symptoms in both “acute phase” and “today.” ** <5 participants experienced these symptoms in “acute phase.” *** <5 participants also experienced these symptoms “today.”

When they reflected on the course of the illness, the participants highlighted the following body functions and symptoms on their drawings as the ones that represented limitations in their daily living: concentration (attention), memory, dyspnoea (respiration functions), lack of energy and drive, fatigue and persistent headache. Figure 1 gives an overview of the symptoms that the participants drew on their timeline, which covered both the acute phase and present time.

The participants referred to their drawings in their dialogue with each other. When interacting with each other in the focus group, several participants recognized symptoms, which they had not inserted in their drawings in the beginning. These symptoms were not added to the drawings (Figure 1).

The thematic analysis of the focus groups generated the following themes: *Persistent symptoms, Balancing activities, Uncertainty Powerlessness* and *Hope*.

#### Persistent Symptoms

The participants described how some of the symptoms they had experienced during their course of illness had never subsided. The symptoms that were widely represented in their dialogues were problems with concentration and memory, lack of energy and drive, fatigue and persistent headaches.

One participant described how lack of energy and drive particularly affected the ability to engage in activities that usually would not be difficult to perform such as hanging clothes from the washing machine or cooking dinner:

“*I haven't had any energy, as I say, I catch myself in sitting down on a chair or in my sofa all the time or (pauses). I actually have lots of plans, and I want to do something, but I constantly catch myself in, like, sitting down.”* (Participant FG 2)

In addition to lack of energy and drive, fatigue was highlighted as a substantial impairment. One participant described this as being the primary obstacle in the path of regaining functioning:

“*Right now it is my main problem preventing me from getting my life back. What is preventing me from getting back to work is fatigue, fatigue and fatigue.”* (Participant FG1)

Another participant elaborated on how the fatigue could be provoked in different ways and how this put up limits for activities:

“*My symptoms, that means fatigue, get worse when I sit and watch television, watch a movie - or when I look too much at the mobile phone or computer screen. So it is not only the physical activity that aggravates… what is it called… my symptoms, it is also mental activity. So, my daily activities are very limited because I simply do not dare to exert myself or exceed the limit. Otherwise, I will be punished with extreme fatigue, and it typically comes late in the evening or the day after.”* (Participant FG1)

The participants also elaborated on how problems with memory and concentration affected their ability in regards to both activities and participation especially in regards to planning activities and executing them. One participant described how planning ordinary activities had become unmanageable to the extent that it results in a large amount of small tasks not being done:

“*And then I stand in the garden looking at the same weed as yesterday. And it becomes a project in my head, something that maybe takes 5 minutes, I'm just looking at it, and can't get it done”* (Participant FG3).

Another participant described how the execution of activities could be affected by disturbances in the surroundings. The participant elaborated on how engaging in more than one activity at a time made it very difficult to concentrate:

“*I can't do two things at once. For example, I can't listen to television and crochet at the same time. Then I might as well sit in a carousel.”* (Participant FG4)

Finally, several participants also struggled with persistent headaches that affected their ability to concentrate and participate in daily activities:

“*There are many things which just make the headache worse. I have a hard time concentrating, I cannot sit in front of a computer screen, it has been really difficult for me to drive a car and to go shopping. I can stand in the store, standing in the queue, and I just want to lie down and sleep. Because, it is like, the headache just shuts down my eyes.”* (Participant FG4)

#### Balancing Activities

The focus groups illuminated participants' experience of constantly balancing the amount and the type of activities to avoid aggravating symptoms. They felt that their symptoms and level of disability fluctuated. They had experienced these fluctuations repeatedly and they connected them to specific activities or to exceeding a limit of tolerance. This limit, however, was not visible to them, and they only knew it had been exceeded when their symptoms worsened. One participant described it as follows:

“*Yeah, the chest pain and headache can also return, if I concentrate too much, if I have too much to do at work. Then the headache also comes. Chest pain can. as late as last week, I felt like.it made me think: “now it really hurts, am I having a heart attack?””*. (Participant FG2)

The participants described that they worried about doing too much, because they had experienced being “punished” by doing too much too fast. Several used the term “dare to” about how they refrained from certain activities or from pushing themselves further toward resuming activities, as this quote illustrates:

“*So now you could do a lot, so now you could get started, and then you just couldn't. It came back like a hammer the next day. For a long period of time it has been like having to walk on that rocking ground. You had to constantly assess - you have to be careful all the time; what should I throw myself into? What do I dare to throw myself into?”* (Participant FG4)

This problem was described by several of the participants in regards to activities and participation related to social activities, job, activities in the household and fitness activities.

#### Uncertainty and Powerlessness

In the attempt of regaining functioning, the participants described themselves in need of guidance to proceed toward their goals. They had a clear picture of what they would like to accomplish but sought specific tools to get there. Uncertainty had emerged from the experience of exceeding limits that aggravated their symptoms, and they were unsure about what to do in different situations.

A participant described this uncertainty in regards to resuming physical activity. Before COVID-19, this participant was used to engage in running and exercising. Trying to resume these activities, the participant experienced symptoms during and after the activities that prevented further progression:

“*We need help to draw a picture of where is it going wrong. What are the things that trigger it? What should your weekly schedule look like? And how can you increase activities next week?”*. (Participant FG4)

Another participant described that it is not an issue of identifying goals but rather an issue of not knowing how to reach them:

“*I can see the lighthouse, but I cannot see the way there. I need someone to help me set the sub-goals I need to proceed.”* (Participant FG2)

A participant added to this that access to specific counseling had been scarce, due to a general lack of knowledge about COVID-19. The participants did not blame the healthcare professionals for this, but described the lack of knowledge as an obstacle in their rehabilitation process:

“*Well that's just that. Nobody knows anything, it doesn't matter where you turn.”* (Participant FG3)

The participants described how these experiences left them powerless. They wished for further clinical assessment and referral to rehabilitation. Some participants described the experience of not getting any better and at the same time struggling to find someone that can help.

In addition, the participants experienced being rejected when they tried to contact the health care system. Specifically, one participant described having tried to get help from municipal rehabilitation professionals several times, but had given up. The participant experienced that there was no clear way into municipal interventions. Stories from other persons with post COVID-19 symptoms who had been offered rehabilitation interventions in other municipalities intensified the participants' frustration:

“*Still, now it doesn't matter. I have soon done it (rehabilitation ed.) myself, but it might have been shortened quite a lot if I had been helped a few months ago. And I think this is a big problem - and I also sense it is different from municipality to municipality*. (Participant FG4)

#### Hope

Despite the difficulties and impairments that the participants were struggling with, their dialogues also reflected hope for the future. This hope was related to how they pictured their lives. The participants related this to wishing for the best and to specific feelings and experiences of regaining abilities, as the following quote illustrates:

“*I take one day at a time and hope for the best. I want to be positive. You* will *get through it and you* will *manage*. (Participant FG2)

They described how the experience of regaining their abilities step by step gave them energy and courage to continue.

“*I have more energy, my mood is better because I feel better. So my general condition feels better. And then the other things will come too. Then the clouds disappear from the sun. And I'm more on my feet, I'm not lying on the couch all day.”* (Participant FG1)

The participants also described that feeling the effect of physical training gave hope and motivation.

Furthermore, their hope was related to the plans they made before they were ill. For some participants this hope was also related to being able to resume or leave work life and still being perceived as an esteemed employee and colleague.

## Discussion

The present study examined functioning and rehabilitation needs among individuals with self-reported disability following COVID-19. The study used a mixed-method design, including PRO, tests of body functions and focus groups with visual drawings.

### Key Findings

The quantitative data showed substantial rehabilitation needs among the participants. On the REHPA scale fatigue, exhaustion, breathing and concentration were reported as the primary causes of loss of functioning. In addition, headaches, balance (motor control), decreased muscle strength and muscle- and joint pain were prevalently reported. The participants also showed low health related quality of life (Median EQ-5D-5L: 60).

There was a large variation on the scales indicating that the degree of rehabilitation needs and quality of life varied within the group.

The tests of body functions showed an overall performance as could be expected in healthy persons in the same age range, with the exception of the 30 s-STS where the median is lower than expected (20).

In the focus groups, the participants described that persistent symptoms affected their daily lives including concentration, memory, lack of energy and drive, fatigue and persistent headaches.

The symptoms fluctuated, and the participants attributed fluctuations to specific activities or to having exceeded their own limits. Consequently, they tried to adjust their activities to avoid aggravating symptoms. The participants further expressed a need for guidance to meet their rehabilitation goals. However, they experienced that their wishes for knowledge, clinical assessment and referral to rehabilitation were not met. This left them with a feeling of powerlessness.

Nevertheless, the participants' dialogues reflected hope for the future. This hope was both related to how they pictured their lives and to experiences of functions, they had already regained.

Drawing on the ICF tool, the themes from the focus groups to some extent fit in to the following categories:

Theme 1) *Persistent symptoms*, particularly in regards to categories within body functions: concentration (b140), memory (b144), lack of energy (b130), fatigue (b1308) and headaches (b28010).
Theme 2) *Balancing activities* in daily living with fluctuating symptoms relates to the described persistent symptoms as well as individual environmental- and personal factors among the participants.
Theme 3) *Uncertainty and Powerlessness* included a need for directional guidance in order to regain functioning and unmet needs regarding further clinical assessment of persistent symptoms, referral to rehabilitation and returning to work. This theme refers to both environmental- and personal factors. Imbedded in this theme is the need for support (e355 Health professionals).
Theme 4) *Hope* included the experiences of recovery and hope for the future, which both refers to personal factors and to individual mental functions (e.g., b1265 optimism and b1266 confidence).

### Integrated Discussion of Qualitative and Quantitative Findings

Both the quantitative and the qualitative data supported that persons who experience persistent symptoms in the aftermath of COVID-19 struggle with a variety of different symptoms and impairments, which may affect quality of life and functioning.

The PCFS and the REHPA Scale both revealed that the participants had rehabilitation needs regarding regaining functioning and that their quality of life may be affected.

Despite these findings, generally, the participants performed well on tests of body functions, which were almost equivalent to a healthy population within the same age range. The majority of the participants scored higher or close to general references on both the HGS strength test (20) and on the 6 MWT (21, 26). Strength as measured by the 30 s-STS was lower than found in a healthy population (20), but higher than reported in large studies in older populations (27).

This could indicate that the chosen tests of body functions were not sensitive enough or did not target the correct issue. Approximately 40% of the participants indicated difficulties with mobility (EQ-5D-5L) and rehabilitation needs related to balance, decreased muscle strength and muscle- and joint pain (REHPA scale) but the tests of body functions do not reflect difficulties to this extent.

The tests of strength and endurance reflect that the primary causes for limitations in this group may lie within other areas of body functions.

On the drawings, concentration, memory, lack of energy and drive and fatigue are the primary symptoms highlighted which echoes the REHPA scale and the themes from the focus groups.

The highlighted symptoms on the drawings are very much consistent with similar studies (4, 23).

Interestingly dyspnoea is an issue that is not reflected in tests of endurance or in the main themes in the focus groups, although it is prominent in both the visual drawings and on the REHPA scale. In the focus groups, dyspnoea is present but very sparsely addressed as a symptom that affects daily living. It is highlighted in the focus groups that the participants struggle to balance their persistent symptoms to avoid aggravating them. Therefore, they are directing a large amount of their focus in this direction. This indicates a possible cause to why dyspnoea is not more prominent in the participants' dialogues. The participants acknowledge that dyspnoea is present—but this symptom and how it affects daily living might simply not be the main problem when balancing other persistent symptoms.

Nevertheless, some participants presented with significant limitations in tests of body functions. Although these limitations seen in tests of body functions are not prominent among the main themes in the focus groups they could still be highly relevant to investigate clinically also for the purpose of tailoring individual rehabilitation interventions.

From themes in the focus groups, it is also clear that access to professional guidance on how to set goals for their rehabilitation and proceed with specific interventions is crucial for the participants. The participants' experience of need for professional guidance is in line with findings from recent studies (5, 7, 28, 29).

Since data for this study was collected, knowledge on post COVID-19 persistent symptoms has steadily increased. In alignment with our study it is firmly stated, that persons who experience persistent symptoms present with a very broad spectrum of symptoms (4, 23).

### Study Strengths and Limitations

The present study has several strengths. The mixed methods design allowed us to examine functioning and disability from different perspectives. While the quantitative data provided information on rehabilitation needs, quality of life and functioning, the qualitative data contributed with rich descriptions of functioning in daily living.

Furthermore, studies from large population- and disease groups were available for comparison of tests of body functions.

The study also has limitations, which need to be taken into account when interpreting the findings. Firstly, in regards to drawing strong statistical conclusions on the quantitative data from the study, a larger population would have been preferable. However, by combining data from both PRO-measures, tests of body functions and qualitative data, the study enhances and deepens our understanding of functioning and rehabilitation needs after COVID-19.

Further, participants may not be representative of the larger population of persons with persistent symptoms after COVID-19: The referral and application procedure may have introduced selection bias. There may also be a healthy volunteer bias, i.e., persons applying for the rehabilitation course may be those with less COVID-19 persistent symptoms. However, all participants in this study present with self-perceived rehabilitation needs, and the study likely contributes to a picture of what clinicians are facing when working with persons with post COVID-19 persistent symptoms.

Finally, the interaction in the focus groups may have drawn participants' attention to a selection of prominent symptoms. This is a weakness, if some participants did not get the opportunity to highlight other symptoms and themes e.g., those regarding symptoms that the other participants did not experience. Individual interviews could have given each participant greater opportunity to elaborate on individual perspectives. However, probing questions were used to assure that participants had the opportunity to mention all relevant issues.

## Conclusion and Clinical Implications

This study highlights that persons with persistent symptoms after COVID-19 may experience a range of symptoms and limitations in their daily living. This points toward a need for individual assessment and guidance to help persons with persistent symptoms regain functioning or cope with possible disability in daily activities and participation. Professional guidance could also support persons with persistent symptoms in dealing with feelings of uncertainty and powerlessness and support hope and goalsetting in the rehabilitation process.

Guidelines on how to conduct evidence-based post COVID-19 rehabilitation interventions have emerged over the course of the pandemic (2, 8, 17), and the present study contributes to this knowledge base by pointing to elements that interventions can include to target the rehabilitation needs of their patients.

At a group level, it would be important to address coping with the common symptoms fatigue, memory and concentration problems. At the same time, the study results point to the importance of an individual approach to rehabilitation, as patients may present with a wide range of symptoms. This calls for a specialized approach based on systematic screening procedures addressing a broad spectrum of potential impairments. This study also indicates that it would be relevant to include components within the mental functions in examining body functions.

When referring people with persistent symptoms after COVID-19 to relevant rehabilitation interventions scales to assess specific rehabilitation needs might be useful for the professionals involved. The scales used in this study represent possible assessment tools that could be used as initial guidance for professionals to prepare and tailor further individual assessments and interventions.

Further research could preferably elaborate on the mechanisms behind COVID-19 persistent symptoms and in more detail examine impairment of mental functions and how these can be assessed.

## Data Availability Statement

The raw data supporting the conclusions of this article will be made available by the authors, upon reasonable request, without undue reservation. Note that all qualitative data are only available in Danish.

## Ethics Statement

The studies involving human participants were reviewed and approved by The Regional Committee on Health Research Ethics for Southern Denmark: Case number 20202000, no. 122. The patients/participants provided their written informed consent to participate in this study.

## Author Contributions

TB, A-DZ, and NR: study design. TB: data collection and analysis and manuscript writing. TB, TM, A-DZ, JR, and NR interpretation and manuscript revisions. All authors contributed to the article and approved the submitted version.

## Conflict of Interest

The authors declare that the research was conducted in the absence of any commercial or financial relationships that could be construed as a potential conflict of interest.

## Publisher's Note

All claims expressed in this article are solely those of the authors and do not necessarily represent those of their affiliated organizations, or those of the publisher, the editors and the reviewers. Any product that may be evaluated in this article, or claim that may be made by its manufacturer, is not guaranteed or endorsed by the publisher.
